# Food Deprivation Affects the miRNome in the Lactating Goat Mammary Gland

**DOI:** 10.1371/journal.pone.0140111

**Published:** 2015-10-16

**Authors:** Lenha Mobuchon, Sylvain Marthey, Sandrine Le Guillou, Denis Laloë, Fabienne Le Provost, Christine Leroux

**Affiliations:** 1 INRA, UMR1313 Génétique Animale et Biologie Intégrative, F-78350, Jouy-en-Josas, France; 2 INRA, UMR1213 Herbivores, F-63122, Saint Genès Champanelle, France; 3 Clermont Université, VetAgro Sup, UMR1213 Herbivores, BP 10448, F-63000, Clermont-Ferrand, France; Nazarbayev University, KAZAKHSTAN

## Abstract

**Background:**

Nutrition affects milk composition thus influencing its nutritional properties. Nutrition also modifies the expression of mammary genes, whose regulation is not fully understood. MicroRNAs (miRNA) are small non coding RNA which are important post-transcriptional regulators of gene expression by targeting messenger RNAs. Our goal was to characterize miRNA whose expression is regulated by nutrition in the lactating goat mammary gland, which may provide clues to deciphering regulations of the biosynthesis and secretion of milk components.

**Methodology/principal findings:**

Using high-throughput sequencing technology, miRNomes of the lactating mammary gland were established from lactating goats fed ad libitum or deprived of food for 48h affecting milk production and composition. High throughput miRNA sequencing revealed 30 miRNA with an expression potentially modulated by food deprivation; 16 were down-regulated and 14 were up-regulated. Diana-microT predictive tools suggested a potential role for several nutriregulated miRNA in lipid metabolism. Among the putative targets, 19 were previously identified as differently expressed genes (DEG). The functions of these 19 DEG revealed, notably, their involvement in tissue remodelling.

**Conclusion/significance:**

In conclusion, this study offers the first evidence of nutriregulated miRNA in the ruminant mammary gland. Characterization of these 30 miRNA could contribute to a clearer understanding of gene regulation in the mammary gland in response to nutrition.

## Introduction

The mammary gland synthesizes and secretes large quantities of milk components during lactation; these include proteins, lactose and lipids which have a considerable effect on the nutritional, technological and sensory properties of milk. Ruminant milk production and composition are linked to both intrinsic and extrinsic factors, such as nutrition [1]. The synthesis and secretion of milk components by mammary epithelial cells involve numerous genes. Interestingly, nutrition affects the expression of genes encoding important factors for milk production in ruminants [2–4]. More precisely, 48 hours of food deprivation applied to lactating goats was shown to cause a drop in milk production and component secretion, associated with the altered expression of 161 genes, including those coding for lipogenic enzymes and major milk proteins [2]. However, the mechanisms underlying the regulation by nutrition of these genes remain poorly documented.

MicroRNAs (miRNA) are small non-coding RNA of ~22 nucleotides in length that principally regulate gene expression by altering mRNA stability or translation. MiRNA are thought to regulate at least ~60% of genes and are involved in all cellular processes [5–8]. Increasing numbers of miRNA are now being identified thanks to the latest advances in deep sequencing which enables access to an overview of the miRNA expressed in a tissue (referred to a miRNome).

However, few studies have reported the role of miRNA in development of the mammary gland [9–13] and in the lactation function [14, 15]. In particular, miRNA have been characterized in the ruminant mammary gland [16–19]. Recent *in vitro* studies have notably revealed potential roles for *miR-103* and *miR-27a* in the regulation of milk fat synthesis in goat mammary epithelial cells [20, 21].

Moreover, miRNA expression and function have been reported to be modulated by diets involving a deficiency or augmented intake [22, 23]. For example, caloric restriction up-regulates the expression of *miR-140-3p* in the epiphyseal growth plate of rats, determined using microarray and RT-qPCR analyses [24]. Ørom *et al*. [25] demonstrated that caloric restriction for six months had a considerable effect on the miRNA profile of the mouse mammary gland, determined by microarray. In addition, the expression of about fifteen known and predicted miRNA was changed in the muscle of monkeys subjected to caloric restriction when compared to those receiving an *ad libitum* diet [26]. Nevertheless, the nutritional regulation of ruminant miRNome is still poorly documented. In particular, in the ruminant mammary gland, no studies offering evidence of the effects of nutrition on miRNA expression have yet been performed.

Therefore, in order to better understand the mechanisms underlying the regulation of mammary gene expression in response to dietary factors, the aim of this study was to obtain an overview of mammary miRNA of goat which received two different diets and to identify miRNA differently expressed in these two conditions. Moreover, we studied the relationship between differently expressed miRNA and genes in the same samples. To our knowledge, this is the first study to have addressed the impacts of nutrition on the expression of miRNA in the mammary gland of ruminants and on lactation.

## Materials and Methods

### Ethics Statement

This study was performed at the experimental unit of INRA Research Center of Theix in 2003. At that time we did not require to submit each animal trail to ethical committee, the institute had its recommendations of the Animal Care as well as the Ethics Committee for Animal Experimentation of our region (Auvergne: CEMEAA number 02) that have been strictly followed. To minimize suffering, all goats were euthanized using captive bolt gun followed by exsanguination at the abattoir of the research center under accreditation number 63 345 001. All samples have been collected once the animals have been slaughtered and not while they were alive.

### Animals and RNA preparation

Twelve Alpine goats at peak lactation (48±2 days post-partum at the beginning of the experiment) from the Lusignan experimental station (France) were chosen based on their homogeneity of their milk yield, number of lactations and their genotype at the α-S1-casein locus which has an impact on milk protein content and indirectly on milk fat content. Goats were fed twice daily an orchard grass hay-based diet for two weeks during the pre-experimental period. Forty-eight hours before slaughtering, six goats were fed *ad libitum* (Control), whereas the six others were food deprived (FD). Milk was collected 15 min before euthanization (S1 Fig). Tissues samples were collected within 30 min after euthanization. All animal manipulations and samples collection were the same as described by Ollier *et al*. [2]. Mammary gland tissues were isolated just after slaughtering, snap frozen immediately and stored at -80°C. RNA were extracted using TRIZol^®^ Reagent (Life Technologies) and further purified using the SV Total RNA isolation system (Promega) to eliminate contaminating genomic DNA. The concentration and quality of RNA preparations were assessed using spectrophotometry (Nanodrop^™^, ND-1000) and 2100 Bioanalyzer Instrument (Agilent). Samples with an RNA Integrity Number (RIN) higher than eight were used. For high throughput sequencing, RNA from four Control goats and six FD goats were used.

### Sequencing library preparation and data processing

The preparation and sequencing of libraries were performed by the IGBMC Microarray and Sequencing Platform (Strasbourg, France). Briefly, 7 μg of total RNA were used to generate small RNA libraries using the TruSeq^™^ Small RNA kit protocol (Illumina). In the first step, RNA adapters were ligated sequentially to each end of the RNA: first the 3′ RNA adapter (5’ TGGAATTCTCGGGTGCCAAGG 3’) that was specifically modified to target small RNA, then the 5′ RNA adapter (5’ GTTCAGAGTTCTACAGTCCGACGATC 3’). Small RNA ligated with 3′ and 5′ adapters were reverse transcribed and PCR amplified (30 sec at 98°C; [10 sec at 98°C, 30 sec at 60°C, 15 sec at 72°C] x 13 cycles; 10 min at 72°C) to obtain cDNA constructs. Finally, acrylamide gel purification of the 140–150 nt amplified cDNA constructs (corresponding to cDNA inserts from small RNA + 120 nt from the adapters) was performed. The libraries were checked for quality and then quantified using a 2100 Bioanalyzer Instrument (Agilent). The libraries were loaded in the flowcell at a concentration of 8 pM and clusters were generated using the Cbot before being sequenced on HiSeq 2500 (Illumina) as single-end 50 base reads, according to Illumina’s instructions.

For analysis, sequences of adapters were removed using Cutadapt [27] and reads were filtered according to their size (17–28 nt). Data analyses were processed using mostly miRDeep2 software [28] as described by Le Guillou *et al*. [19]. The cleaned sequences were clustered into unique reads and then mapped to the reference Goat genome (CHIR_1.0, http://goat.kiz.ac.cn/GGD/, [29]). Precursors and miRNA were identified using the miRDeep2 core module, miRDeep2.pl. Potential miRNA datasets were created by adding known miRNA in goat (miRBase v21) to miRNA associated with predicted precursors with a miRDeep2 score ≥0. This number scored for the likelihood for a miRNA to be a real precursor according to the combined of energetic stability, positions and frequencies of read with Dicer processing. This score is comprised between 10 and -10, so a score ≥0 has been chosen representing because it allowed the discovery of a maximum of miRNA minimizing the rate of false positives. Then, the same operation was performed to create a data set of potential precursors. The quantifier.pl miRDeep2 module was then used to map unique reads, the set of potential miRNA and all known miRNA (miRBase v21) on the set of potential precursors enabling the annotation of miRNA. The quantification results produced by the quantifier.pl module were then filtered with a custom perl script parse_miRDeep2_outputs.pl (https://mulcyber.toulouse.inra.fr/projects/bioinfoutils/) to eliminate any redundancy between known and predicted miRNA. RNA sequencing data were deposited in the Gene Expression Omnibus (GEO): GSE61025.

### Quantitative RT-PCR

Seven miRNA were chosen for RT-qPCR validation among known miRNA which expression was affected by the food deprivation and based on their level of expression; *miR-99a-5p* (TaqMan^®^ ID 006254_mat, Applied Biosystems), *miR-126-3p* (TaqMan^®^ ID 008451_mat), *miR-140-3p* (TaqMan^®^ ID 471823_mat), *miR-222-3p* (TaqMan^®^ ID 000525), *miR-223-3p* (TaqMan^®^ ID 002295), *miR-204-5p* (TaqMan^®^ ID 000508), *miR-409-3p* (TaqMan^®^ ID 002332). Reverse transcription was achieved on 10 ng of total RNA using the TaqMan^®^ MicroRNA Reverse Transcription kit (Applied Biosystems, Foster City, CA, USA), according to the manufacturer’s instructions. In the thermal cycler (StepOne+, Applied Biosystems, Foster City, CA, USA), each 15 μL RT reaction followed 30 min at 16°C, 30 min at 42°C and 5 min at 85°C. Then, 1.3 μL of miRNA-specific cDNA from the reaction were amplified using TaqMan^®^ Small RNA Assays (Applied Biosystems, Foster City, CA, USA) according to the manufacturer’s instructions. Amplification was performed at 95°C for 10 min, followed by 40 cycles of 95°C for 15 sec and 60°C for 1 min. All miRNA levels were normalized to the values of *U6* snoRNA [30, 31] and the results expressed as fold changes of threshold cycle (Ct) values relative to the control using the 2^-ΔΔCt^ method [32].

### Statistical analysis

For sequencing data, a comprehensive description of miRNA expression patterns was first of all made using a specific principal component analysis (PCA). Each goat is represented by a point, each colour corresponds to a diet and goats which have received the same diet are connected to their barycentre and an inertia ellipse.

Statistical analysis to determine differential expression was performed with R version 3.0.2 (R Development Core Team, 2013, http://www.R-project.org/) using the Bioconductor package DESeq2 version 1.0.17 [33]. DESeq2 utilizes a negative binomial distribution to model read counts per miRNA and then implements a method to normalise the counts. This normalisation procedure uses the library median of the ratios between the read count and the geometric mean of each miRNA as a scaling factor for each library. Fold changes were estimated using an empirical Bayes shrinkage procedure.

This procedure helps to moderate the broad spread in fold changes for genes with low counts, while it has negligible effect on genes with high counts. Since hypothesis tests are performed for miRNA-by-miRNA differential analyses, the p-values obtained need to be adjusted to correct for multiple testing. However, procedures to adjust p-values in order to control the number of false positives found often lead to a loss of power to detect truly differentially expressed miRNA because of the large number of hypothesis tests performed. To reduce the impact of such procedures, the filtering method described by Rau *et al*. [34] was used to remove genes that appeared to generate an uninformative signal. This method identifies a filtering threshold that maximizes so-called filtering similarity among replicates. Tests for differential expression were only applied to miRNA whose maximum count across all ten samples was higher than its threshold. This method was implemented under the Bioconductor HTSFilter package, version 1.0.0. The threshold value was found to be equal to 28. The p-values were adjusted for multiple testing using the Benjamini and Hochberg method [35], and those with an adjusted p-value <0.1 were considered to be significant.

For RT-qPCR analyses, data are represented as log base 2 of the fold change between Control and FD goats. The data were analysed using the Mann-Whitney test performed using R version 3.0.2 (R Development Core Team, 2013, http://www.R-project.org/).

### Analysis of targeted pathways

Putative targets for differently expressed miRNA were predicted with a high degree of accuracy based on DIANA-microT-CDS version 5.0 [36] (http://diana.imis.athena-innovation.gr/). Targets of predicted miRNA were searched using TargetScan Custom release version 5.2 [37] (http://www.targetscan.org/). Putative targeted pathways were investigated through the use of QIAGEN’s Ingenuity Pathway Analysis (IPA, QIAGEN Redwood City, www.qiagen.com/ingenuity).

## Results and Discussion

### Global description of mammary miRNomes in lactating goats

Ten libraries were constructed from the mammary gland of four Control and six FD goats using the Illumina/Solexa technology. A mean of about 14.2 and 16.4 million reads were obtained from the Control and FD libraries, respectively (Table 1). After removing sequencing adapters and filtering reads by their size, 6.8 and 8.5 million reads of 17–28 nt were obtained for the Control and FD libraries, respectively. The reads were mapped to the goat genome (CHIR 1.0), and clustered in 67,971 and 75,474 unique sequences, on average, in the Control and FD libraries, respectively. Using the goat precursors reported in miRBase version 21 (v21), 1,070 precursors were first of all predicted by miRDeep2 for Control and FD goats. Then, using all matures from miRBase v21, it was possible to identify 1,804 miRNA. This step allowed the detection of 539 known miRNA, corresponding to 422 miRNA known in goat and 117 miRNA not yet described in the goat but known in other species. In addition, 1,265 predicted miRNA were characterized, corresponding to miRNA never described in any species.

**Table 1:** Summary of sequencing data.

	Control^a^	FD^b^
Raw reads	14,214,146	16,415,279
Cleaned reads^c^	13,787,000	15,681,069
Sized reads^d^	6,838,797	8,464,633
Sized and unique sequences processed	215,663	204,005
Reads mapped^e^	5,784,949	7,278,339
Unique sequences corresponding to mapped reads	67,971	75,474

^a^means of data for the 4 Control goat libraries

^b^means of data for the 6 FD goat libraries

^c^sequencing adapters removed

^d^17-28 nt size filter, used by the miRDeep2 software

^e^reads with at least one and at most five reported alignments, used by the miRDeep2 software.

**Fig 1:**
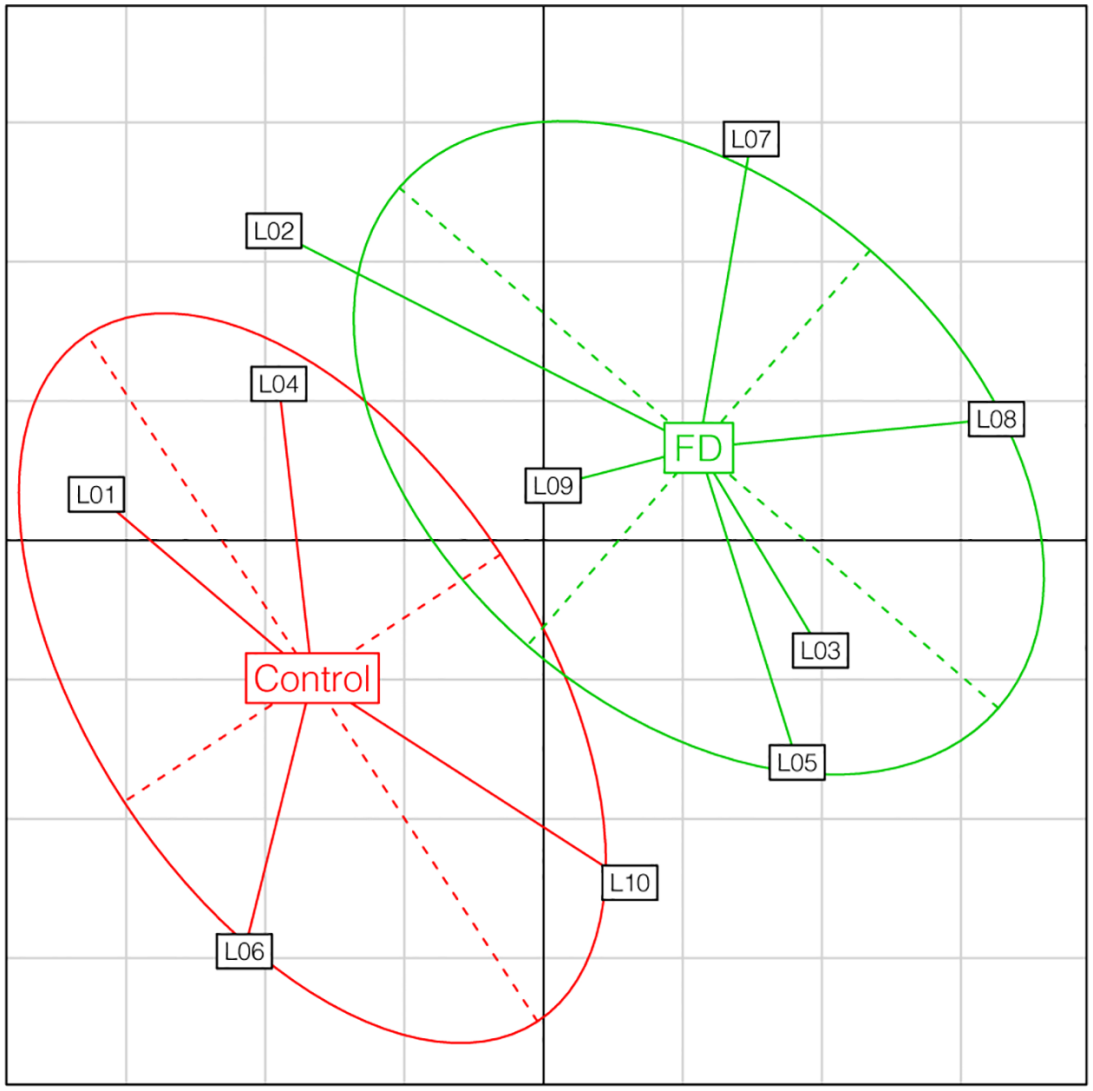
Principal component analyses of miRNA in Control and FD goats. L0X: represent 1 library, FD: Food deprived. Analyses were performed on miRNA for libraries according to the diet received by the goats. The two different diets are labelled within their 95% inertia ellipse (Red: Control; Green: FD).

By performing expression pattern analysis using PCA, the ten libraries appeared to be clearly discriminated according to the treatments applied to the goats (Fig 1).

Using HTSFilter [34], a statistical threshold was applied and reduced the 1,804 miRNA detected to 470, 343 were known and 127 predicted (S1 Table). At this step, lists of miRNA were conserved between the different nutritional conditions.

### Identification of miRNA differentially expressed in the mammary gland of goats after food deprivation

To identify miRNA significantly affected by food deprivation, a differential analysis was performed. Using the DESeq2 package, of the 470 miRNA remaining after the HTSFilter analysis, 30 miRNA were found to be differently expressed between Control and FD goats, of which 14 were up-regulated and 16 were down-regulated (Table 2). Among the potentially nutriregulated miRNA, 19 were known miRNA and 11 were predicted (Table 2 and S2 Table; S2 Fig). Among these 30 potentially nutriregulated miRNA pointed out by high-throughput sequencing, the expression of seven miRNA have been studied by RT-qPCR analysis on 12 goats (six Control and six FD) (S3 Fig) with the aim to reinforce the sequencing data. Probably due to the difference of sensibility and normalization, only the expression of *miR-204-5p* was found significantly affected when analysed using RT-qPCR. Nevertheless, the orientation of regulation of all miRNA tested was in accordance with data obtained by sequencing data. It should be noted that nutritional intervention studies aim to measure low changes in gene expression. In these conditions, it has been suggested that PCR should not be considered as validation tool but as complementary technology [38]. The authors concluded that PCR approach allowed picking up the expression of genes that were not measurable on high throughput technology but also vice versa, and that both techniques have their own (dis)advantages and specificities. For less pronounced changes, both technologies may be useful as complementation rather than validation.

**Table 2:** MiRNA whose expression was affected by food deprivation in lactating goat mammary gland.

miRNA name	Family	Sequence	Control*	FD*	Fold change (FD/control)	P-value adjusted
*Known miRNA*						
***miR-6119-5p***		AGAGGTAAAAAATTGATTTGACT	19,082	14,396	0.77	0.07
***miR-126-3p***	mir-126	TCGTACCGTGAGTAATAATGC	17,218	22,489	1.28	0.06
***miR-660-5p***	mir-188	TACCCATTGCATATCGGAGCTGT	7,698	6,144	0.81	0.09
***miR-99a-5p***	mir-10	AACCCGTAGATCCGATCTTGT	5,533	7,709	1.35	0.06
***miR-451-5p***	mir-451	AAACCGTTACCATTACTGA	4,957	6,855	1.33	0.1
***let-7c-5p***	let-7	TGAGGTAGTAGGTTGTATGGTT	4,934	6,427	1.28	0.06
***miR-140-3p***	mir-140	ACCACAGGGTAGAACCACGGAC	3,798	4,888	1.27	0.09
***miR-409-3p***	mir-154	GAATGTTGCTCGGTGAACCCC	1,954	1,159	0.66	0.06
***miR-125b-3p***	mir-10	ACAAGTCAGGCTCTTGGGACC	1,918	2,496	1.27	0.06
***miR-222-3p***	mir-221	AGCTACATCTGGCTACTGGGTCTC	812	1,408	1.47	0.09
***miR-204-5p***	mir-204	TTCCCTTTGTCATCCTATGCCT	541	1,113	1.8	0.00
***miR-99a-3p***	mir-10	CAAGCTCGCTTCTATGGGTCTGT	472	615	1.27	0.08
***miR-196a-5p***	mir-196	TAGGTAGTTTCATGTTGTTGG	413	663	1.47	0.06
***miR-494-3p***	mir-154	TGAAACATACACGGGAAACCTCT	311	197	0.67	0.05
***miR-223-3p***	mir-223	TGTCAGTTTGTCAAATACCCCA	245	552	1.65	0.06
***miR-188-5p***	mir-188	CATCCCTTGCATGGTGGAGGG	117	81	0.73	0.09
***miR-671-5p***	mir-671	AGGAAGCCCTGGAGGGGCTGGAGG	73	50	0.73	0.09
***miR-541-5p***	mir-541	AAAGGATTCTGCTGTCGGTCCCACT	29	15	0.64	0.08
***miR-223-5p***	mir-223	TGTGTATTTGACAAGCTGAGTTG	17	34	1.6	0.06
*Predicted miRNA*						
***chr12_17655-3p***		CCCGGGTTTCGGCACCA	611	337	0.66	0.09
***chr19_26739-5p***		GAGGGTTTGGGTTTGGTCGTGGGA	508	289	0.67	0.09
***chr3_4386-5p***		ATAGTTCATTCAGGTTTTTCTG	420	227	0.66	0.09
***chr27_34538-5p***		AAAGTTCATTCAGGTTTTTCTG	412	224	0.66	0.09
***chr3_4386-3p***		GAAGAATCTGAATGAACATTT	253	152	0.66	0.05
***chr23_30758-5p***		GGGGATGTAGCTCAGTGGTAGA	133	68	0.66	0.09
***chr9_13534-5p***		GTACATGATGACAACTGGCTC	83	113	1.33	0.06
***chr12_18027-5p***		GAAAGGTTCATTTGGGTTTTT	56	97	1.51	0.08
***chr22_29775-3p***		ATCAGAGTCACGGCACCA	37	16	0.59	0.05
***chr3_3319-5p***		GAAAGTTTGTTTGGGTTTTTC	39	13	0.63	0.08
***chr4_6064-5p***		CCCGATCTTGTCTGATC	29	10	0.63	0.08

*Mean of normalized read counts.

The seed region of each miRNA is underlined. A positive log2 fold change means the corresponding miRNA is more highly expressed in FD goats than in Controls and vice versa. P-values were adjusted using the Benjamini-Hochberg [35] correction at 10%.

Looking at the abundance of the 30 nutriregulated miRNA, two known miRNA (*miR-126-3p* and *miR-6119-5p*) were very highly expressed with normalized read counts greater than 10,000, while seven (*let-7c-5p*, *miR-99a-5p*, *miR-125b-3p*, *miR-140-3p*, *miR-409-3p*, *miR-451-5p* and *miR-660-5p*) were highly expressed and represented by normalized read counts of between 1,000 and 10,000 (Table 2). In addition, seven known (*miR-99a-3p*, *miR-188-5p*, *miR-196a-5p*, *miR-204-5p*, *miR-222-3p*, *miR-223-3p* and *miR-494-3p*) and six predicted miRNA (*chr3_4386-3p*, *chr3_4386-5p*, *chr12_17655-3p*, *chr19_26739-5p*, *chr23_30758-5p* and *chr27_34538-5p*) were expressed at moderate levels with normalized read counts of between 100 and 1,000, and three known (*miR-223-5p*, *miR-541-5p* and *miR-671-5p*) and five predicted (*chr3_3319-5p*, *chr4_6064-5p*, *chr9_13534-5p*, *chr12_18027-5p* and *chr23_30758-5p*) were weakly expressed with normalized read counts below 100. This last category may have a lesser impact in cells because of their weak expression.

Interestingly, the expression of both matures of the same precursor (*mir-99*, *mir-223* and *chr3_4384*) were affected by nutrition. This case has only ever been reported once for miR-9/9*, whose expression was dramatically decreased after maternal supplementation with retinoic acid in the rat embryo spina bifida model [39]. The regulation may occur on the promoter of miRNA gene leading to deregulate both -5p and -3p strands. In all other cases in the literature, only one strand of the precursor has been found to be affected by nutrition. These observations suggest that two different regulatory mechanisms may exist. In this case, the regulation may happen on the stability of one or the other strand leading to the deregulation of only one strand by nutrition which seems to be the main mechanism in our study. These two different mechanisms of regulation require further investigations.

Otherwise, two clusters, defined as miRNA genes with an inter-distance ≤10 kb [40], were altered by food deprivation. Indeed, *miR-494-3p*, *miR-409-3p* and *miR-541-5p* are members of the greatest and conserved miRNA cluster located on goat chromosome 21. These three miRNA were down-regulated by food deprivation and their expressions were well correlated with a high mean Pearson correlation coefficient of 0.8. Similarly, *let-7c-5p* and *mir-99*, which clustered on the goat’s X chromosome, were up-regulated in FD goats and displayed a strong mean Pearson correlation coefficient of 0.8. These results are consistent with the previously reported co-regulation of clustered miRNA, where they were transcribed as a long polycistron [41].

Among the nutriregulated miRNA, five (*miR-126-3p*, *miR-140-3p*, *miR-223-3p*, *miR-409-3p*, *miR-451-5p*) have previously been described as being affected by caloric restriction in others species and tissues. The expressions of *miR-223-3p* and *miR-409-3p* were increased, whereas that of *miR-451-5p* was decreased in skeletal muscle of monkeys receiving a restricted diet [26]. Conversely, *miR-409-3p* was down-regulated in the blood of mice receiving about 60% of their normal diet, whereas the expression of *miR-451-5p* was increased [42]. Most notable was the fact that the expressions of *miR-409-3p* (down-regulated in FD) and *miR-451-5p* (up-regulated in FD) appeared to be sensitive to caloric restriction. This apparent discrepancy between findings may have been due to the type of restriction, and probably to the tissues and species studied. In addition, the expressions of *miR-126-3p* and *miR-140-3p*, which were up-regulated in our study, were down-regulated in the epiphyseal growth plate of Sprague-Dawley rats by 60% food restriction for 10 days [24]. Although this nutritional model appears to be the closest to ours, deprivation in our case was much more drastic because the animals received no nutrients at all. Finally, among the predicted miRNA, the *chr3_4386-5p* and *chr27_34538-5p* sequences were found to be almost similar except at position 2, suggesting that these miRNA may belong to the same miRNA family. In addition some nutriregulated miRNA belonged to the same family. Indeed, *miR-409-3p* and *miR-494-3p* belonged to the *mir-154* family and *miR-188-5p* and *miR-660-5p* to the *mir-188* family (Table 2). Interestingly, it is known that members of a miRNA family are designed to execute similar biological functions [43]. Therefore, nutriregulated miRNA sharing the same family may have linked roles in the lactating mammary gland.

### Putative functions of nutriregulated miRNA

**Fig 2:**
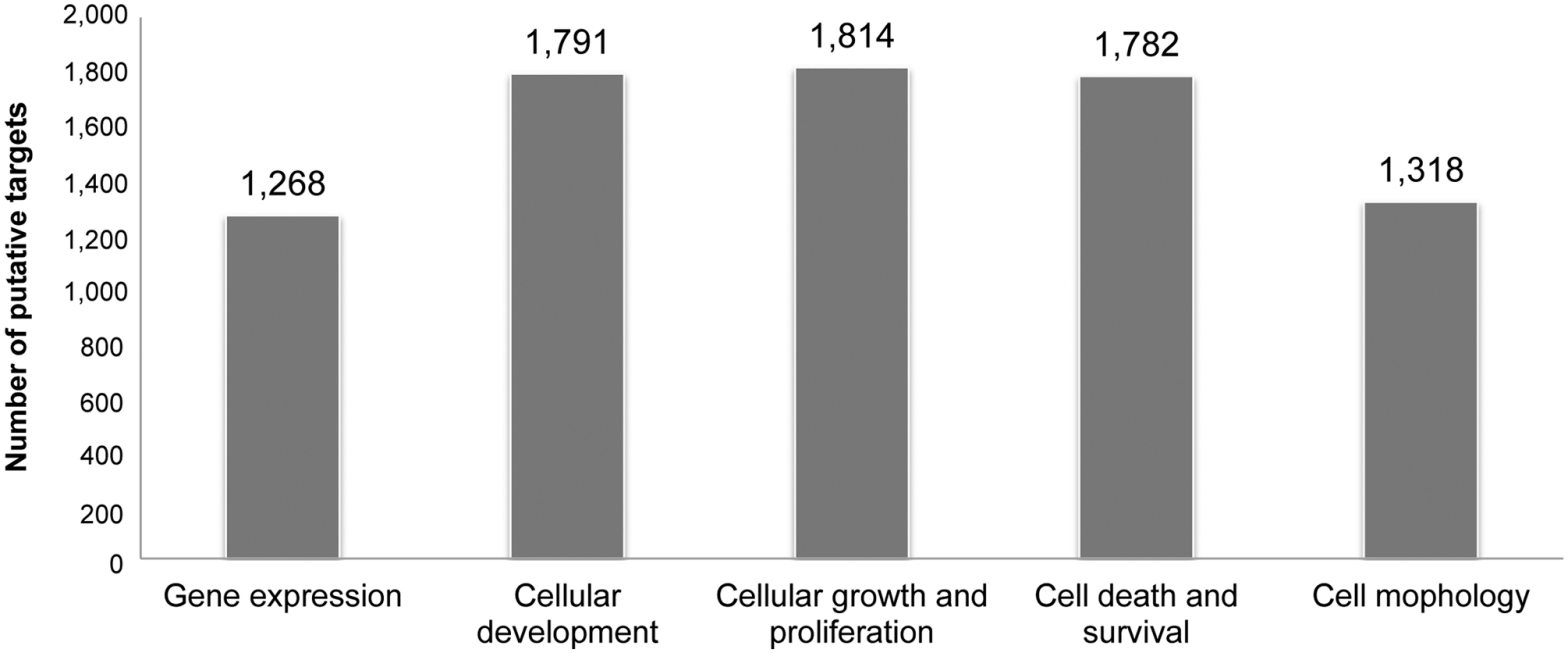
Molecular and cellular functions of potentially targeted genes by the 30 nutriregulated miRNA. Data were analysed through the use of QIAGEN’s Ingenuity Pathway Analysis (IPA^®^, QIAGEN Redwood City, www.qiagen.com/ingenuity).

Among the 30 miRNA that were regulated in our model, only a few have previously been described in the mammary gland. In the mouse mammary gland, *miR-126-3p* may inhibit cell proliferation; it may also regulate the expression of *CSN2* coding for β-casein, one of the major milk proteins, and PGR (ProGesteron Receptor) protein [44]. In addition, *miR-99a-5p* has been described as a modulator of the TGF β pathway affecting epithelial-to-mesenchyme transition in normal mouse mammary gland [45]. Therefore, in order to investigate the functional role of the 30 nutriregulated miRNA in the mammary gland, target gene predictions were performed based on miRNA/mRNA interactions using Diana-microT v5.0 for known miRNA and TargetScan Custom v5.2 for predicted miRNA. Because no predictive tools have so far been designed for ruminants, conservation between goat miRNA and humans was checked, particularly concerning the seed region that determines miRNA interactions with mRNA targets. Thus, 7,129 different putative targets for the 30 nutriregulated miRNA were identified. Investigating the molecular and cellular functions of these targets using Ingenuity Pathway Analysis revealed that “gene expression”, “cellular development” and “cellular growth and proliferation” were the three pathways most significantly targeted (Fig 2). These findings suggest that food deprivation might lead to a change in gene expression through the actions of miRNA linked to cellular growth and proliferation, as well as the remodelling of mammary cells, as has been observed in this organ in the event of nutrient restriction [46].

In addition, some putative targets of nutritregulated miRNA in this study were associated with involution in the mammary gland; these included factors promoting involution such as *CASP3*, *IL6* and *STAT3*, or factors inhibiting involution such as *BCL2* [47, 48] (S3 Table). Markers of autophagosome formation, *MAP1LC3B2* and *ATG7*, were also found to be potentially targeted by *miR-188-5p* and *miR-223-3p*, respectively. These observations were in line with the drop in milk production and component synthesis previously observed in FD goats [2].

### Potential involvement of nutriregulated miRNA in the mammary biosynthesis and secretion of milk components

#### Potential involvement in mammary lipid metabolism

**Fig 3:**
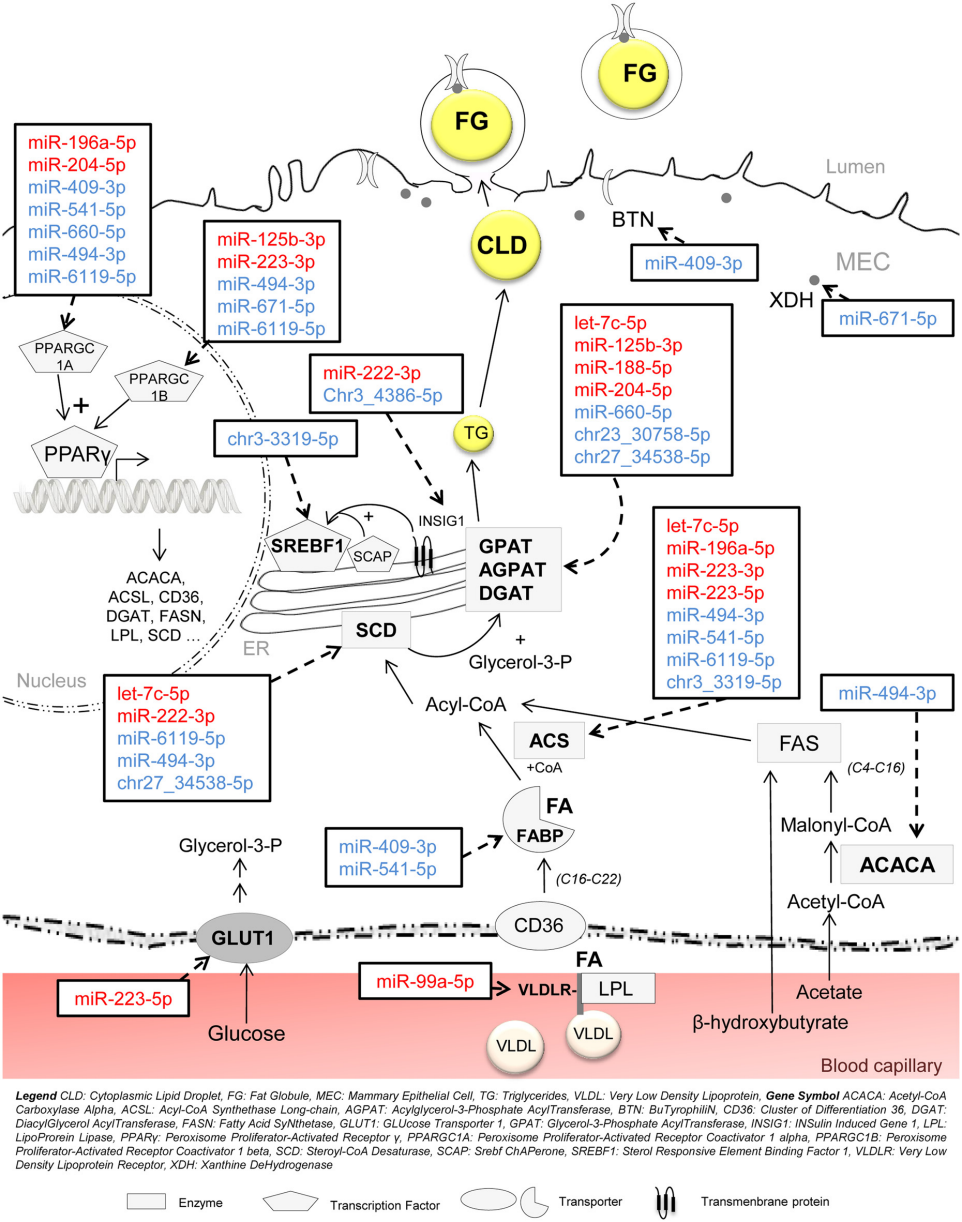
Potential involvement of nutriregulated miRNA in milk fat synthesis. In red, miRNA which expression was up-regulated in FD goat mammary glands compared with Controls, while those whose expression was down-regulated are in blue.

**Fig 4:**
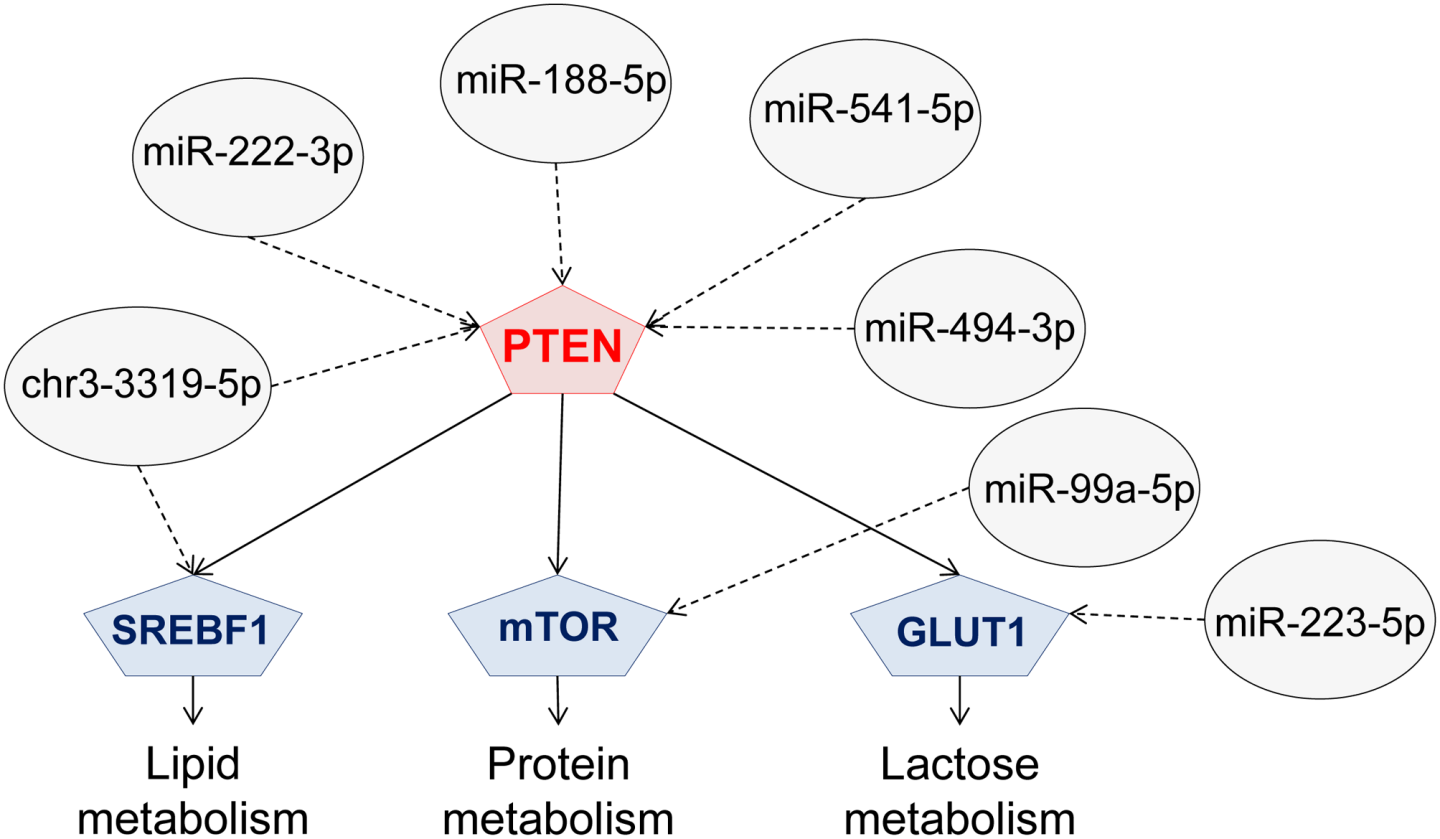
Potential contribution of nutriregulated miRNA to regulating PTEN and associated networks. GLUT1: GLUcose Transporter 1, mTOR: mechanistic Target Of Rapamycin, PTEN: Phosphatase and TENsin homolog, SREBF1: Sterol REsponsive Binding Factor 1.

Of all the 7,129 putative targets of nutriregulated miRNA, particular efforts were made to identify those involved in lipid metabolism. Very interestingly, nutriregulated miRNA may act in fatty acid availability (*VLDLR*), synthesis (*ACACA*), transport (*FABP*), activation (*ACS*) and desaturation (*SCD*), triglyceride synthesis (*AGPAT*) and in genes coding for proteins associated with the milk fat globule membrane (*BTN*, *XDH*) (Fig 3) [49]. Several nutriregulated miRNA also target transcription factors such as the SREBF1 and PPARγ cofactors, *PPARGC1A* and *PPARGC1B* that govern the expression of essential lipogenic enzymes in the mammary gland (Fig 3). Moreover, other genes related to milk lipid metabolism, such as *INSIG2* (INSulin_Induced Genes 2) which codes for an endoplasmic reticulum protein that blocks the processing of SREBP, is predicted to be targeted by *chr12_18027-5p* [50]. Elsewhere, *miR-204-5p* may also target *LPIN2* (LiPIN 2), recently characterized in the bovine mammary gland [49] and involved in triacylglycerol accumulation [51]. The gene coding for FADS1 (Fatty Acid DeSaturase 1), which plays a role in triacylglycerol synthesis [52], is potentially targeted by *miR-671-5p*. Furthermore, *miR-125b-3p*, *miR-494-3p* and *chr3_3319-5p* may conjointly target *ABCA1* (ATP-Binding Cassette subfamily A member 1) which has been hypothesized to play a role in the transfer, storage and removal of cholesterol in the mammary gland [53]. Finally, five nutriregulated miRNA: *miR-222-3p*, *miR-188-5p*, *miR-541-5p*, *miR-494-3p* and *chr3_3319-5p*, may target *PTEN* (Phosphatase and TENsin homologue) (Fig 4). In particular, *miR-494-3p* binding has been confirmed experimentally in bronchial epithelial cells [54]. The role of *PTEN* has been characterized in dairy cow mammary epithelial cells (DCMECs), revealing that it plays a crucial role in the viability and proliferation capacity of cells, as well as in the secretion of β casein, triglycerides and lactose. In particular, *PTEN* may regulate the expression of key lactation-related pathways such as *PPARγ*, *SREBF1*, *mTOR*, *PRLR* and *GLUT1*, suggesting a pleiotropic role for *PTEN* in the lactation process [55].

Among the predicted miRNA, *chr3_3319-5p* appeared to be of great interest. Indeed, *chr3_3319-5p* potentially targets numerous genes involved in mammary metabolism. Its seed region is similar to that of *hsa-miR-561-3p*. This miRNA has not yet been reported in either the goat or cattle, but its identical seed region with *hsa-miR-561* suggests that this miRNA is part of the *mir-561* family, even though the function of this family is not yet known in the mammary gland (Fig 4, S4 and S5 Tables).

Taken together, these observations highlight a potentially synergistic action of miRNA in regulating mammary metabolic pathways, and offer perspectives for the study of new types of regulation in the lactating mammary gland in response to dietary treatments. Those miRNA which expression was affected by nutrition may modulate that of the genes responsible for fatty acid metabolism, lipid droplet formation and/or milk fat globule secretion at the same time, and thus consequently have a crucial role to play in the synthesis and secretion of milk components. In particular, *miR-409-3p* and *miR-494-3p* may target numerous genes involved in lipid metabolism and its regulation. It was notable that they belong to the same cluster and the same family, *mir-154*, which could be of considerable interest in future studies.

#### Potential involvement in the metabolism of mammary protein and lactose

Proteins are also a principal constituent of milk and are synthesized by mammary epithelial cells during lactation. Amino acids are supplied to the mammary epithelial cells by a system of amino acid membrane transporters [56]. Among the genes putatively targeted by nutriregulated miRNA, several amino acid transporters were identified (Table 3). The availability of amino acids in the mammary gland is important for the regulation of translation, and also appears to be one of the major limitations affecting milk protein synthesis [57], and nutriregulated miRNA may regulate this availability. Caseins (αs1, ɑs2, β andκ), the major milk proteins, are phosphorylated in mammary epithelial cells. This phosphorylation is involved in aggregation of the four caseins in micelles before their secretion into milk. Despite this key role, the phosphorylation step in mammary epithelial cells is still poorly documented. Interestingly, *miR-222-3p*, *miR-409-3p*, *miR-541-5p*, *miR-6119-5p*, *miR-660-5p*, *chr23_30758-5p* and *chr3_4386-5p* potentially target several genes coding for casein kinases 1 and 2 alpha and gamma (S4 Table). In addition, *miR-204-5p* potentially targets *ELF5* (E74-like factor 5) which belongs to the JAK-STAT signalling pathway and is essential for the expression of milk-related genes, particularly those coding for major milk proteins [58, 59]. Above all, *miR-99a-5p* was found to potentially target *mTOR* (mechanistic Target Of Rapamycin) and its binding site has been confirmed experimentally using the Luciferase Reporter Assay in breast cancer cell lines [60]. The regulation of protein synthesis in all mammalian tissues is under the control of *mTOR*, and especially the translation step. In addition, *mTOR* may also have a function in milk protein synthesis [59] (S4 Table), suggesting a potential role of *miR-99a-5p* in controlling this process.

**Table 3:** Amino acid transporters potentially targeted by nutriregulated miRNA.

miRNA	Gene	Protein	Associated transport system	Description	*References*
miR-223-5p, miR-541-5p, miR-671-5p	SLC1A1	GLT-1;EAAT1	X^-^_AG_	Na+-dependent system for anionic amino acids	[61, 62]
miR-223-5p	SLC1A3	GLAST;EAAT1	X^-^_AG_	Na+-dependent system for anionic amino acids	[61]
miR-6119-5p	SLC1A4	ASCT1-SATT	ASC	Na+-dependent transporter, particularly linear dipolar amino acids (L-alanine, L-serine, L-cysteine)	[56]
miR-409-3p, miR-541-5p, chr3_3319-5p	SLC6A6	Taut	System Gly	Na+-Cl^-^-dependent system specific to β-amino acids	[61]
miR-671-5p	SLC7A1	CAT-1	y+	Na+-independent system specific to cationic amino acids	[63]
miR-126-3p	SLC7A5	LAT1	L	Na+-dependent electroneutral transport mechanism for neutral amino acids	[59]
miR-196a-5p, miR-223-3p	SLC7A8	LAT2	L	Na+-dependent electroneutral transport mechanism for neutral amino acids	[62]
miR-409-3p, miR-494-3p	SLC15A2	PEPT2	H+-peptide cotransporter 2	Proton-peptide electrogenic transporter	[64]
miR-671-5p	SLC36A1	PAT1		Proton/amino acid symporter	[59]
let-7c-5p, miR-409-3p, miR-494-3p	SLC38A2	SNAT2	System A	Na+-dependent system for amino neutral acids	[62]

Putative targets were predicted from DIANA microT v5.0 [36] for known miRNA and Custom Target Scan v5.2 [37] for predicted miRNA.

Regarding differentially expressed miRNA targeting the SLC (Solute Carrier) family (S4 Table), *miR-223-5p* was found to target *SLC2A1* (GLUT1). Glucose is the primary precursor for lactose synthesis, the main milk carbohydrate. In the mammary gland of lactating cows, *GLUT1* mRNA and protein were shown to be strongly expressed whereas they were barely detectable in dry cows [65]. In addition, GLUT1 protein represents about half of glucose transporters in the Golgi membranes of lactating rat epithelial cells [66]. Consequently, *miR-223-5p* may have a role in regulating glucose uptake and lactose synthesis, a hypothesis which requires further investigation.

### Putative targets differently expressed in FD goats

**Fig 5:**
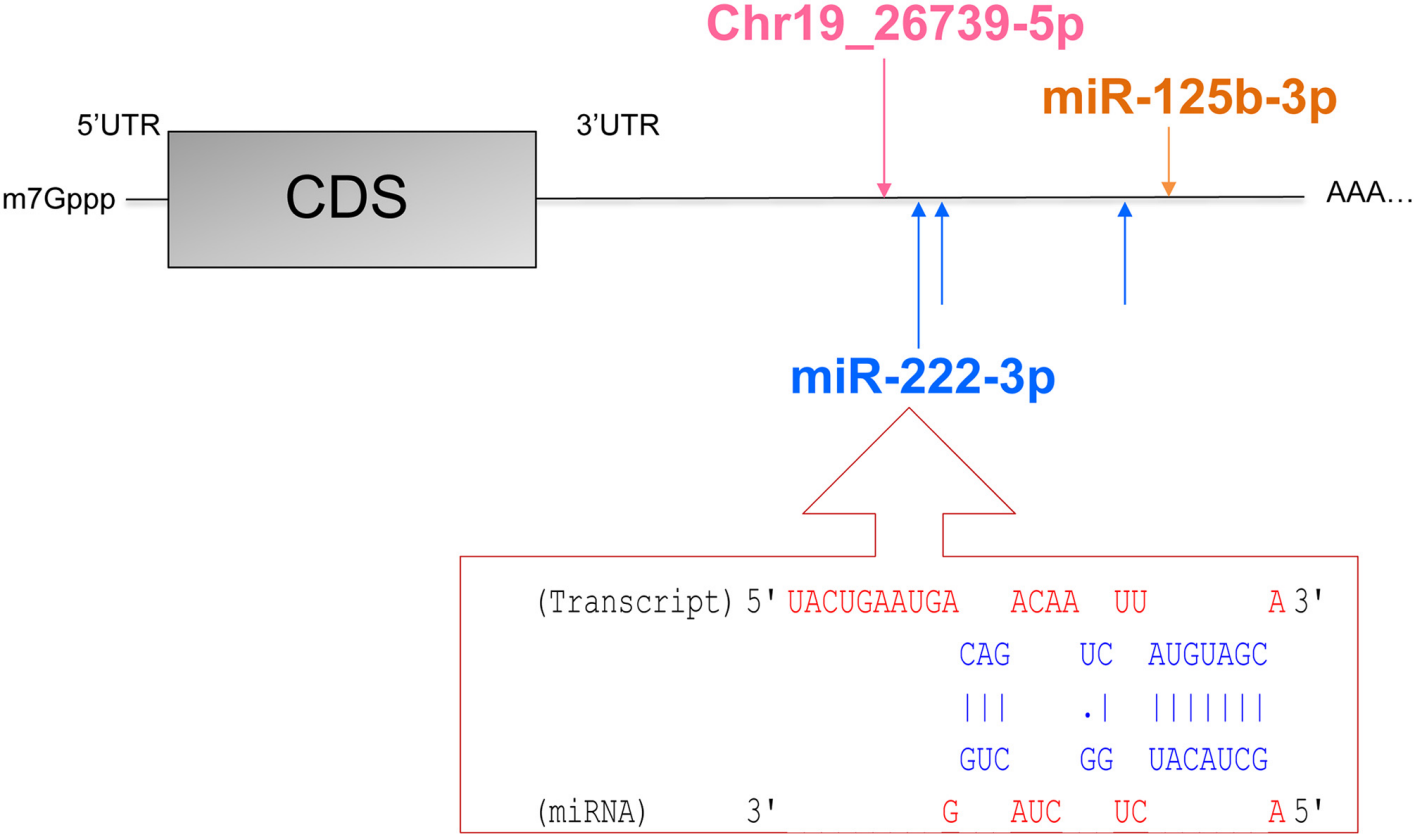
Nutriregulated miRNA binding sites on *ESR1* mRNA. Analyses were performed using DIANA microT v5.0 [36] for miR-125b-3p and miR-222-3p and Custom Target Scan v5.2 [37] for chr19_26739-5p. The interaction between 3’UTR of ESR1 and the seed region of miR-222-3p of one binding site is described in the box.

Of all the putative targets predicted for nutriregulated miRNA, 47 were among the genes previously identified as being significantly affected (named DEG) by food deprivation using a microarray technique [2] (S5 Table). One to six miRNA were predicted to targets these 47 DEG. In addition, when the way of variations in DEG and nutriregulated miRNA was taken account, 19 DEG were found to be potentially targeted by nutriregulated miRNA, including 18 down-regulated DEG which might be targeted by one to three up-regulated miRNA (Table 4) one up-regulated DEG (*CD24*) might be targeted by *chr3_3319-5p* which was down-regulated in FD goats. By investigating the cellular and molecular functions of these 19 DEG, “cellular death and survival”, “cellular morphology” and “cell-to-cell interaction” were found to be among the five most targeted functions (data not shown). This suggests that the deregulation of miRNA may explain the phenotype of mammary tissue remodelling previously hypothesized in FD goats [2]. Among the DEG that may be targeted by three nutriregulated miRNA, *ESR1* is a transcription factor that regulates genetic programming of cell cycle progression and growth in the mammary gland. The knockout of *ESR1* in mice suggests its crucial role in alveologenesis during lactation [67]. Interestingly, *ESR1* was down-regulated by food deprivation and potentially targeted by *miR-125b-3p*, *miR-222-3p*, and *chr19_26739-5p*, and up-regulated in the mammary gland of FD goats. In addition *miR-222-3p* has been confirmed as directly targeting *ESR1* in breast cancer cell lines [68]. Its binding site (Fig 5) is conserved among different species, including cattle and goats. These observations suggest that the up-regulation of *miR-222-3p* by food deprivation may be responsible for the down-regulation of *ESR1*.

**Table 4:** Differently expressed genes potentially targeted by nutriregulated miRNA.

miRNA	Differently Expressed Genes
**let-7c-5p**	ABCC5, ANKFY1, DCBLD1, DCUN1D3, DPAGT1, LEPREL2, PLAGL2
**miR-99a-3p**	ZCCHC14
**miR-125b-3p**	ESR1
**miR-140-3p**	PLAGL2
**miR-204-5p**	IRF2BP2, KCNH8, PHLDB1, TSPAN31
**miR-222-3p**	ESR1, INSIG1
**miR-223-3p**	ANKFY1, ARFIP1, MYO1B, PLAGL2, ZCCHC14
**miR-223-5p**	EFHC1
chr3_3319-5p	**CD24**
**chr9_13534-5p**	ESR1, ZCCHC14
**chr12_18027-5p**	RAD9A, TMEM2

In bold, miRNA or DEG whose expression are upregulated by the food deprivation. ABCC5: ATP-binding cassette, sub-family C (CFTR/MRP), member 5, ANKFY1: Ankyrin repeat and FYVE domain-containing protein 1, ARFIP1: Arfaptin-1, CD24: Signal transducer CD24 precursor, DCBLD1: Discoidin, CUB and LCCL domain-containing protein 1 precursor, DCUN1D3: DCN1, defective in cullin neddylation 1, domain containing 3, DPAGT1: UDP-N-acetylglucosamine-dolichyl-phosphate N-acetylglucosaminephosphotransferase, EFHC1: EF-hand domain-containing protein 1, ESR1: Oestrogen receptor alpha, INSIG1: Insulin-induced gene 1 protein, IRF2BP2: Interferon regulatory factor 2 binding protein 2, KCNH8: Potassium voltage-gated channel subfamily H member 8, LEPREL2: Prolyl 3-hydroxylase 3 precursor, MYO1B: Myosin Ib, PHLDB1: Pleckstrin homology-like domain family B member 1, PLAGL2: Zinc finger protein PLAGL2, RAD9A: Cell cycle checkpoint control protein Rad9A, TMEM2: Transmembrane protein 2, TSPAN31: Tetraspanin-31, ZCCHC14: Zinc finger CCHC domain-containing protein 14.

Furthermore, 11 nutriregulated miRNA (Table 4) were found to potentially target DEG in which three predicted miRNA *chr3_3319-5p*, *chr9_13534-5p* and *chr12_18027-5p*. Of the 11 nutriregulated miRNA that could target DEG, *let-7c-5p*, *miR-223-3p* and *miR-204-5p* may target seven, five and four DEG, respectively (Table 4). Notably, *miR-204-5p* and *miR-223-3p* are nutriregulated miRNA with the highest fold change in high throughput sequencing. In addition, *miR-223-3p* may target five DEG, three of which (*ANKFY1*, *PLAGL2* and *ZCCHC14*) encode for zinc finger proteins that bind DNA, suggesting that this miRNA may indirectly regulate gene transcription. Consequently, the deregulation of *miR-204-5p* and *miR-223-3p* by food deprivation may have a significant impact in the mammary gland through the regulation of DEG.

### Co-localization of nutriregulated miRNA with QTL associated with traits

Quantitative Trait Loci (QTL) have been associated with economically important traits such as health through the detection of clinical mastitis (somatic cell score) as well as milk production and composition (lactose, protein and fat) in livestock, particularly cattle [69]. However no QTL are yet available in the goat. Using conservation between species, cow miRNA expressed during lactation and conserved in the goat have been located in cattle QTL associated with milk production and composition (personal communication). Among the 30 miRNA nutriregulated in the present study, *mir-671* was located in QTL linked to somatic cells on bovine chromosome 4 (Table 5). It is also located in a QTL region associated with both milk protein yield and milk fat percentage and content. In addition, *mir-140* is located in QTL associated with the somatic cell score and milk yield on bovine chromosome 18. Furthermore, *mir-409*, *mir-494* and *mir-541* belonging to a large and conserved cluster in mammals have also been located in QTL associated with milk yield and milk fat percentage and content on bovine chromosome 21. As well as having many putative targets involved in lipid metabolism (Fig 3, S4 Table), *miR-494-3p* and *miR-409-3p* are also positioned in QTL regions associated with milk fatty acids, thus rendering these two miRNA good candidates to study their involvement in the regulation of milk lipid synthesis. The co-localization of nutriregulated miRNA with QTL associated with milk composition may provide clues to their role in the regulation of milk traits.

**Table 5:** Nutriregulated miRNA which bovine equivalent is located in QTL associated with milk production and composition.

QTL	*Associated with milk components*		*Associated with mammary tissue health*
Bovine chromosome	Fat percentage and content	Protein yield	Somatic cell score
BTA 4	mir-671	mir-671	mir-671
BTA 18			mir-140
BTA 21	mir-494, mir-541, mir-409a		

Conservation between nutriregulated and bovine miRNA was checked and their localization in QTL (CattleQTLdb, http://www.animalgenome.org/cgi-bin/QTLdb/BT/) associated with milk was investigated.

## Conclusion

The findings reported here highlight for the first time using high throughput miRNA sequencing, 30 known or predicted miRNA in the ruminant mammary gland which expression seems to be nutriregulated. Among these 30 potentially interesting miRNA, members of clusters and similar families were found. Five of them have already been reported as being affected by nutrition in other species and tissues. By investigating the genes they might target, some nutriregulated miRNA appear to be good candidates for further studies in mammary gland biology. For example, *miR-494-3p*, located in QTL associated with milk fat in cows, may be preponderant for milk lipid synthesis, notably through the regulation of *PTEN*, and in targeting *mTOR*, *miR-99a-5p* may regulate milk protein synthesis. The links between miRNA and DEG previously identified by transcriptomic assays show that 19 DEG may be targeted by nutriregulated miRNA. These observations highlighted two miRNA, *miR-204-5p* and *miR-223-3p*, whose expression was the most markedly affected by food deprivation and which may target several DEG, suggesting important roles for both miRNA in the nutritional regulation of gene expression in the mammary gland. Nevertheless, further studies are necessary to support our results and to decipher the function of these miRNA in mammary epithelial cells.

MiRNA offer new insights to understand the impact of nutrition on the mammary gland and may help to clarify mammary gene regulation in order to modulate milk quality via nutrition. The nutrition model of food deprivation used in this study was a large challenge, so further studies should be performed with a diet that could be applied by livestock farmers in order to identify the nutriregulation of miRNA under breeding conditions.

## Supporting Information

S1 Fig Milk production, fat, protein and lactose in Control and FD goats. Data were extracted from [2] and were analysed with a Mann-Whitney test (n = 6). **: p<0.01.d: day.
(TIF)

S2 Fig Structure of predicted precursors whose miRNA are differently expressed in FD versus Control goats. Putative targets were predicted from DIANA microT v5.0 [36] for known miRNA and Custom Target Scan v5.2 [37] for predicted miRNA.
(TIF)

S3 Fig Quantitative RT-PCR validation of NGS data. The expression of miRNA was normalized with the expression of U6 in Control and FD goats. The relative abundance of the miARN is represented with boxplots (n = 6).
(TIF)

S1 Table miRNA expressed in lactating goat mammary glands. (XLSX)

S2 Table Characteristics of nutriregulated predicted miRNA. (XLSX)

S3 Table Example of putative targets involved in mammary involution. (XLSX)

S4 Table Selected putative targets involved in mammary metabolism. (XLSX)

S5 Table Differently expressed genes potentially targeted by nutriregulated miRNA. (XLSX)
