# Supplementary figures and images for: Food Deprivation Affects the miRNome in the Lactating Goat Mammary Gland
Source: PLoS One. 2015 Oct 16;10(10):e0140111. doi: 10.1371/journal.pone.0140111 (PMC4608672; doi:10.1371/journal.pone.0140111)

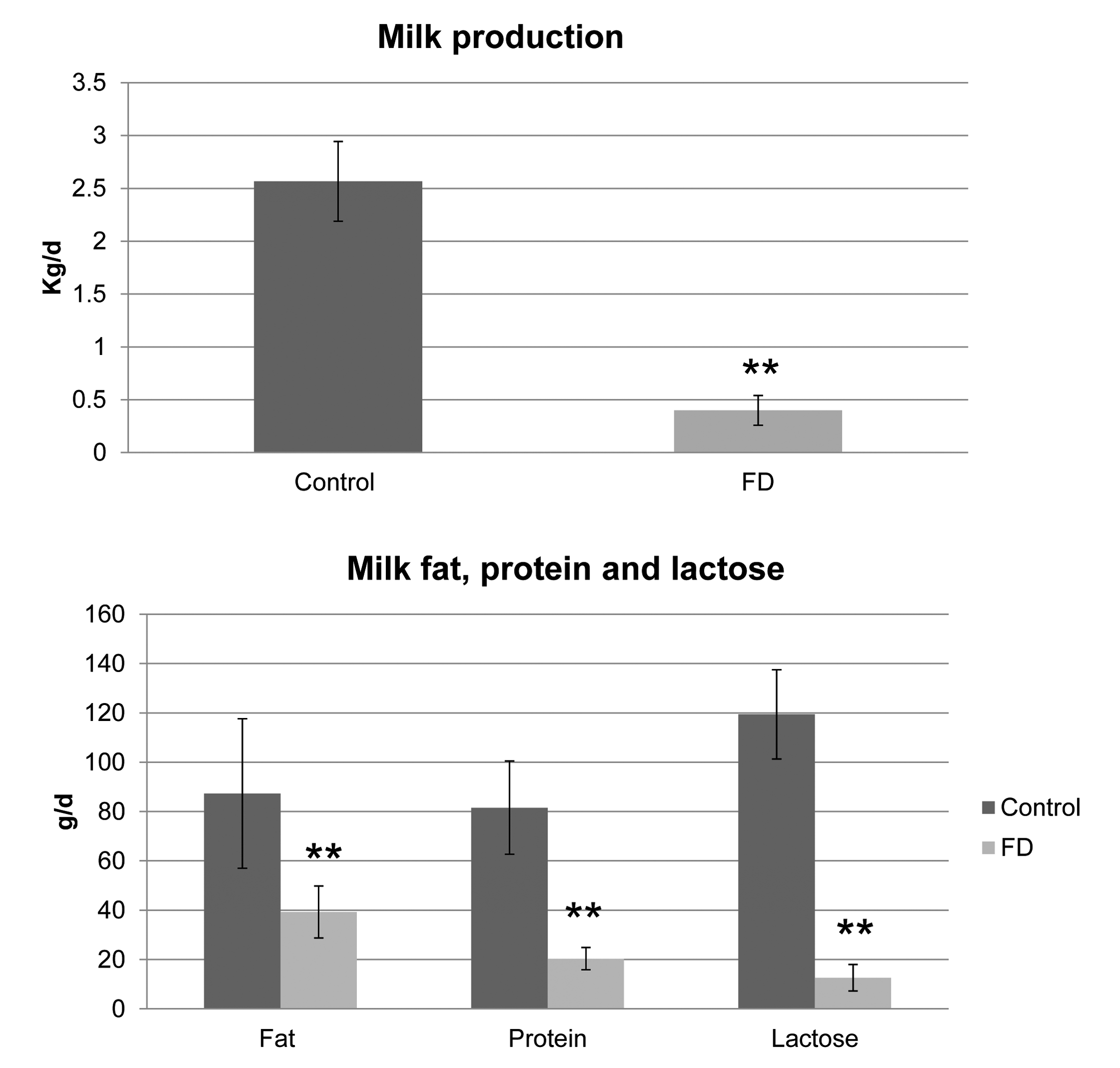

Supplement: S1 Fig — Data were extracted from [2] and were analysed with a Mann-Whitney test (n = 6). **: p<0.01.d: day. (TIF) [file pone.0140111.s001.tif]

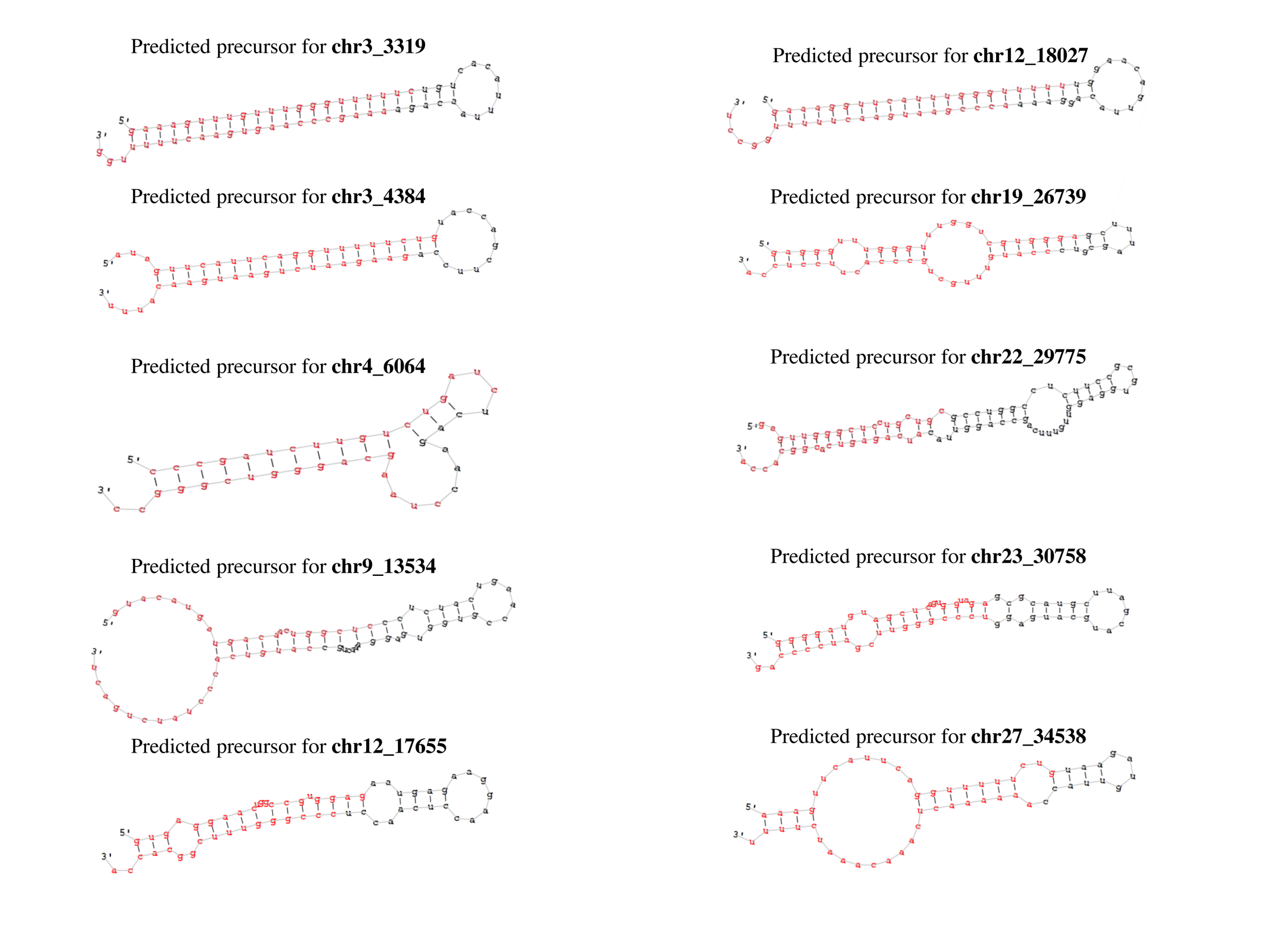

Supplement: S2 Fig — Putative targets were predicted from DIANA microT v5.0 [36] for known miRNA and Custom Target Scan v5.2 [37] for predicted miRNA. (TIF) [file pone.0140111.s002.tif]

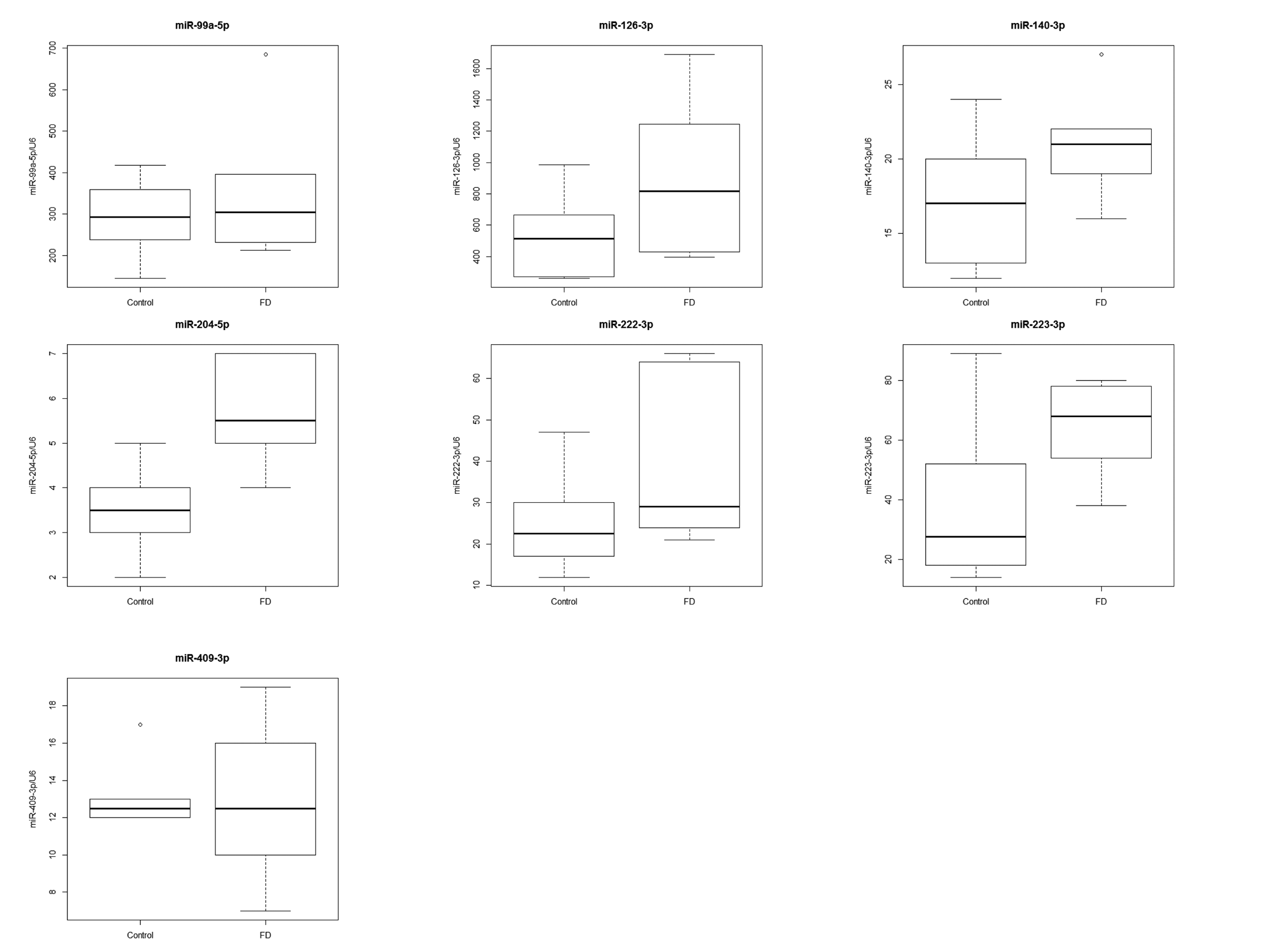

Supplement: S3 Fig — The expression of miRNA was normalized with the expression of U6 in Control and FD goats. The relative abundance of the miARN is represented with boxplots (n = 6). (TIF) [file pone.0140111.s003.tif]
